# Corneal backscatter as a biomarker for edema severity in Fuchs endothelial corneal dystrophy: a cross-sectional study

**DOI:** 10.1186/s40662-026-00483-3

**Published:** 2026-04-30

**Authors:** Maximilian Friedrich, Hyeck-Soo Son, Lasha-Giorgi Turava, Maximilian Hammer, Louise Blöck, Lizaveta Chychko, Timur Mert Yildirim, Gerd Uwe Auffarth, Victor Aristide Augustin

**Affiliations:** Department of Ophthalmology, David J. Apple International Laboratory for Ocular Pathology and International Vision Correction Research Centre (IVCRC), University of Heidelberg, Im Neuenheimer Feld 400, 69120 Heidelberg, Germany

**Keywords:** Fuchs endothelial corneal dystrophy, Subclinical corneal edema, Scheimpflug tomography, Densitometry, Corneal backscatter, Edema severity

## Abstract

**Background:**

Fuchs endothelial corneal dystrophy (FECD) is characterized by progressive endothelial dysfunction and corneal edema. Reliable objective biomarkers for grading disease severity are currently evolving. Corneal backscatter may reflect edema-related changes in corneal transparency and could serve as an additional objective measure of FECD severity across different edema stages. The purpose of this study was to evaluate the potential of corneal backscatter as a biomarker for severity of FECD.

**Methods:**

In this retrospective, observational, single-center cross-sectional study, 99 eyes of 67 patients with FECD were divided into three groups depending on the presence of clinical, subclinical or no corneal edema. The corrected distance visual acuity (CDVA) was obtained and Scheimpflug tomography was performed. Subclinical corneal edema was diagnosed if more than one of the following criteria were present in Scheimpflug tomography: loss of regular isopachs, displacement of the thinnest point of the cornea, and focal posterior corneal surface depression. Clinical corneal edema was diagnosed by slit-lamp biomicroscopy. The primary outcome was the difference in total corneal backscatter in the central 2-mm zone. Secondary analyses were the correlation of corneal backscatter to CDVA, tomographic parameters such as central corneal thickness (CCT) and thinnest corneal thickness (TCT), as well as their diagnostic accuracy and predictive potential to differentiate between edema severity.

**Results:**

The total central corneal backscatter was significantly higher among eyes with high edema severity (*P* < 0.05). Differences in corneal backscatter between subgroups diminished with increasing depth of corneal layer and towards the periphery. The anterior (Marginal R^2^ = 0.260; *P* < 0.001) and total (Marginal R^2^ = 0.208; *P* < 0.001) corneal backscatter in the central 2-mm zone correlated well with CDVA. Corneal backscatter showed a higher correlation with CDVA than CCT (Marginal R^2^ = 0.198; *P* < 0.001) and TCT (Marginal R^2^ = 0.096; *P* = 0.004). Central corneal backscatter in the anterior 120 µm demonstrated moderate diagnostic accuracy for distinguishing subclinical corneal edema from no edema (threshold = 31.2 GSU; AUC = 0.73) or clinical edema (threshold = 34.9 GSU; AUC = 0.76).

**Conclusions:**

Our study corroborates that corneal backscatter values increase with increasing corneal edema stage and therefore present a potential adjunctive biomarker to assess FECD severity in addition to edema status and clinical gradings.

## Background

Fuchs endothelial corneal dystrophy (FECD) is the most common corneal dystrophy with significant impact on visual acuity, contrast sensitivity and straylight [1–7]. There are different classification systems available for grading its severity. The oldest classification system is the Krachmer scale which grades the number and distribution of corneal guttae as well as the presence of clinical corneal edema using slit-lamp biomicroscopy [8]. In the modified Krachmer, Grade 6 clinical corneal edema is present and often impedes the visual function significantly [9]. However, in earlier stages, additional diagnostic criteria are necessary for classifying the disease stage and visual relevance of the disease [10]. More recently, Sun et al. published three criteria to diagnose a subclinical corneal edema using corneal tomography [11]. The presence of a subclinical tomographical edema was shown to significantly reduce the visual function and induce higher-order aberrations compared to FECD patients without the presence of a subclinical corneal edema [2, 3]. However, in some cases there may be intra- or inter-observer disagreement due to the partly subjective nature of these criteria [12]. This implicates the need for additional, objective parameters to detect subclinical corneal edema more reliably even in borderline cases.

With increasing improvement of clinical outcomes after endothelial keratoplasty in recent years, corneal surgeons may advise surgical intervention even in earlier stages of FECD (e.g., subclinical edema stage). In fact, it was shown that an earlier Descemet membrane endothelial keratoplasty (DMEK) yields significantly better visual results compared to an intervention in the later stage with clinical corneal edema [13]. This functional difference may be attributed to corneal fibrosis caused by preoperative chronic clinical edema, which may be identified as corneal backscatter [13]. This is supported by previous studies which have shown that corneal backscatter correlates to conventional FECD stages such as the modified Krachmer scale and progressive visual impairment [14–16]. Thus, we hypothesize that corneal backscatter may serve as a valuable objective biomarker for FECD staging to help refine edema stage differentiation and support surgical decision-making.

The purpose of this study was to compare corneal backscatter values depending on the edema severity and to analyze their correlation to visual acuity compared to established diagnostic parameters in FECD patients.

## Methods

We included a total of 99 eyes of 67 patients with FECD in this retrospective, observational, single-institution cross-sectional study. Inclusion criteria were: (1) examination between 2020 and 2025 by the same consultant (V.A.A.) in the cornea department of the tertiary hospital; (2) examinations performed with sufficient quality; (3) FECD without any another endothelial disease, especially pseudophakic bullous keratopathy; (4) eyes without previous ocular surgery other than uncomplicated cataract surgery or other ocular comorbidities. In total, 108 patients were screened and 41 patients were excluded as they did not fulfill the inclusion criteria in both eyes. Of the remaining 67 patients, 35 eyes were excluded due to ocular comorbidities (n = 20 eyes) or insufficient scan quality (n = 15 eyes).

This study was approved by the Institutional Review Board/Ethics Committee (ID: S-621/2021) at the Ruprecht-Karls University Heidelberg, Germany, and performed in accordance with the tenets of the Declaration of Helsinki. Informed consent was obtained from all participants.

### Measurements

All eyes underwent slit-lamp biomicroscopy and the FECD stage was graded according to the modified Krachmer scale [17]. We included eyes with modified Krachmer Grade 5 (confluent guttae over 5 mm in diameter and no clinically evident corneal edema) or modified Krachmer Grade 6 with clinically visible corneal edema. The corrected distance visual acuity (CDVA) was measured for each eye in the morning under photopic conditions (320 cd/m^2^) using an electronic 5-letter per-line chart at 5-m test distance.

All eyes underwent Scheimpflug tomography (Pentacam AXL, Oculus Optikgeräte, Wetzlar, Germany) and the 4 Maps Refractive output as well as the Corneal Densitometry output were obtained. In accordance to the classification published by Sun et al. [11], subclinical tomographical corneal edema in eyes without clinical corneal edema was diagnosed in each eye if two or three of the following criteria were present: loss of regular parallel isopachs, displacement of the thinnest point of the cornea from the center and focal posterior surface depression of the cornea. All eyes were graded individually by two masked observers (M.F. and V.A.A.) and interrater variances were resolved by the grading of a third masked observer (H.-S.S.). Inter-observer agreement was assessed by the percentage of agreement between both observers. If one or no criterion was present, the eye was classified as ‘without corneal edema’.

The 4 Maps Refractive tomography output provided measurements of central corneal thickness (CCT) and thinnest corneal thickness (TCT) as well as asphericity and flat keratometry of the corneal back surface. From the Corneal Densitometry output, total corneal backscatter was obtained, along with backscatter values segmented by surface area and corneal layer. In Pentacam AXL, the corneal layer is divided into the anterior 120 µm, the posterior 60 µm and the middle layer (called central) between the anterior and posterior layer. In the frontal plane, the densitometry is reported in corneal annuli (0–2 mm, 2–6 mm, 6–10 mm, 10–12 mm) in addition to the value of total corneal diameter (0–12 mm). The densitometry values were expressed in gray scale units (GSU), where 0 GSU represented the lowest backscatter and 100 GSU the highest.

### Statistical analyses

We performed the statistical analyses with R statistical software (Version 4.2.2, R Foundation for Statistical Computing, Vienna, Austria) using the R packages “clusrank”, “lmerTest” and “MuMIn” [18, 19]. We performed clustered Wilcoxon rank-sum tests using the Datta-Satten method [20] for comparison of metric variables to account for the inclusion of both eyes of a patient in some cases. The primary outcome was the differences in total corneal backscatter in the central 2-mm corneal zone between all three groups. As three significance tests were performed regarding the primary outcome, the significance level of 0.05 was adjusted to 0.017. The sample size calculation to find a significant difference in total corneal backscatter in the central 2-mm corneal zone with an anticipated baseline mean of 31.7 ± 8.3 GSU [13] for the edema group and minimum detectable difference of 5 GSU (α = 0.05; β = 0.8; enrollment ratio 1:1) resulted in at least 30 eyes for each group. The descriptive statistics are presented as median [interquartile range].

As an exploratory secondary analysis, the correlation between CDVA and different corneal backscatter parameters as well as common objective parameters in FECD assessment (CCT, TCT) were evaluated. For correlation analysis, we performed mixed effects models with the patient ID as a cluster variable to account for the inclusion of both eyes of a patient in some cases. We reported the correlation coefficient β, the Marginal R^2^ as well as Cohen’s f^2^ to describe the power of correlation with f^2^ ≥ 0.02 representing a small, f^2^ ≥ 0.15 a moderate, and f^2^ ≥ 0.35 a large effect size, respectively. Additionally, we performed a diagnostic accuracy analysis to distinguish subclinical corneal edema from no edema and clinical edema using the included tomographic parameters. The threshold values were selected according to the highest Youden index. In addition to the univariate analysis, a multivariate analysis using a generalized estimating equation with stepwise backward-selection was performed clustered by patient to predict the edema group allocation. As predictors, the most promising variables were included: Anterior, central and posterior densitometry of the central 2-mm zone, flat keratometry and asphericity of the corneal back surface, TCT, and CCT. To account for the different scales of the predictors, all values were standardized.

## Results

The group with clinical corneal edema consisted of 31 eyes (28 patients), the group with subclinical corneal edema consisted of 37 eyes (25 patients), and the group without edema consisted of 31 eyes (21 patients). The study eyes’ characteristics are summarized in Table 1. The inter-observer agreement regarding the classification was high (92.9% agreement; n = 92 eyes) and discrepancies (n = 7 eyes) were resolved by a joint assessment of the authors.

**Table 1:** Characteristics of all study eyes, classified by edema severity

Group	Gender (n)		Eye (n)		Age (years)	Pseudophakic proportion	CDVA (logMAR)	Corneal thickness (µm)	
	Female	Male	Right	Left				Central	Thinnest
No corneal edema	13	18	15	16	63 [23]	48.4%	0.1 [0.25]	554 [57]	552 [57]
Subclinical corneal edema	17	19	19	17	71 [17]	70.3%	0.2 [0.18]	598 [51]	588 [45]
Clinical corneal edema	19	13	17	15	71 [12]	83.9%	0.4 [0.27]	643 [87]	602 [88]

Descriptive statistics of ratio-scaled variables are presented as median [interquartile range]

*LogMAR* = logarithm of the minimum angle of resolution

### Corneal backscatter depending on edema group

**Fig. 1:**
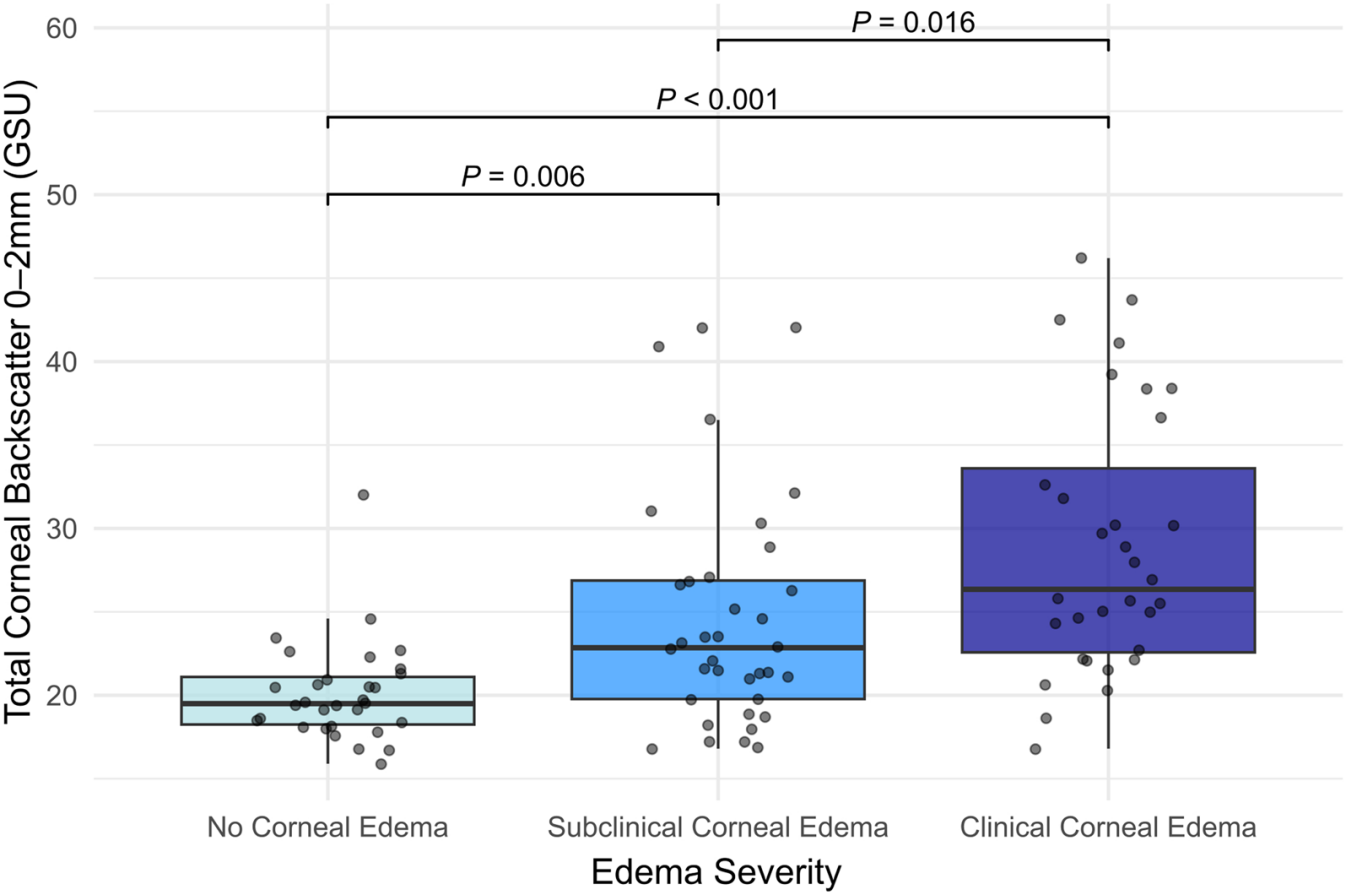
Total corneal backscatter in the central 2-mm zone depending on edema severity. Significance bars indicate the *P* value of the performed clustered Wilcoxon rank-sum tests. GSU, gray scale units

The total corneal backscatter values in the central 2-mm zone differed significantly between edema groups (*P* < 0.017, Fig. 1). The group without corneal edema showed significantly lower total backscatter values (19.50 GSU [2.85]) than the group with subclinical corneal edema (22.85 GSU [7.1]; *P* = 0.006) and clinical corneal edema (26.35 GSU [11.03]; *P* < 0.001). Additionally, the total corneal backscatter values in the central 2-mm zone differed significantly between the groups with subclinical and clinical corneal edema (*P* = 0.016). As age distribution in the three edema groups could have confounded the backscatter value, we performed a correlation analysis between age and corneal backscatter using a mixed-effects model. There was no statistically significant relationship between age and backscatter of any corneal layer and annuli (all *P* < 0.05).

**Fig. 2:**
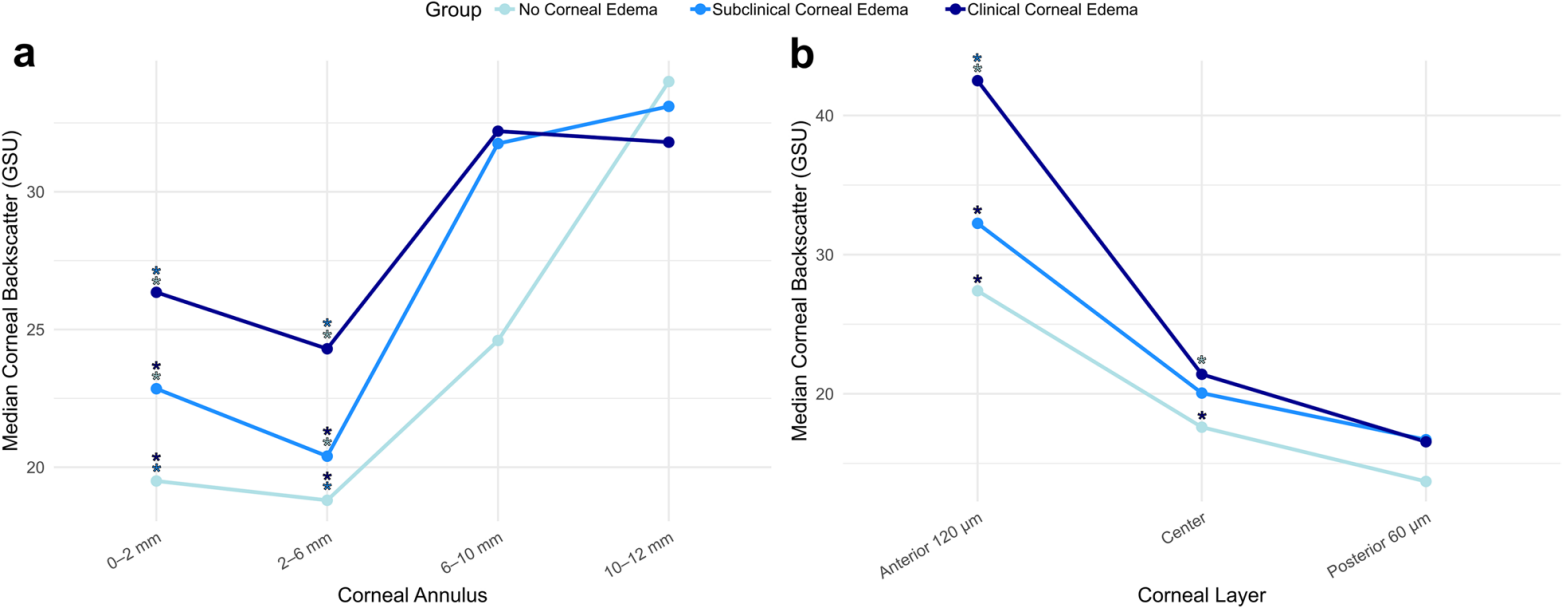
Relationship between corneal backscatter and annulus (**a**) and layer (**b**) depending on edema severity in Fuchs endothelial corneal dystrophy. Coloured asterisks indicate explorative statistical significance (*P* < 0.05) of the respective group value to the group of the corresponding color. GSU, gray scale units

The median values of corneal backscatter in all zones and layers in regard to edema severity are shown in Table 2. The central 2-mm zone as well as the anterior 120 µm layer showed the most striking difference in corneal backscatter between edema severity grades. Towards the more peripheral corneal annuli, the median backscatter values became more similar (Fig. 2a). A similar trend was observed with increasing depth of corneal layer (Fig. 2b).

**Table 2:** Corneal backscatter values depending on edema severity divided into corneal layer and zone and the exploratory *P* values of clustered Wilcoxon rank-sum test comparisons

Corneal backscatter parameter (GSU)		No corneal edema	Subclinical corneal edema	Clinical corneal edema	*P* value No edema vs. Subclinical edema	*P* value No edema vs. Clinical edema	*P* value Subclinical edema vs. Clinical edema
Total thickness	0–2 mm annulus	19.50 [2.85]	22.85 [7.10]	26.35 [11.03]	**0.006**	**< 0.001**	**0.016**
	2–6 mm annulus	18.80 [2.60]	20.40 [7.67]	24.30 [6.85]	**0.026**	**< 0.001**	**0.038**
	6–10 mm annulus	24.60 [11.55]	31.75 [13.63]	32.20 [17.35]	0.144	0.052	0.723
	10–12 mm annulus	34.00 [11.35]	33.10 [12.25]	31.80 [19.28]	0.673	0.586	0.838
	Total diameter	23.30 [5.35]	25.85 [10.48]	29.10 [10.05]	0.117	**0.005**	0.218
Anterior 120 µm	0–2 mm annulus	27.40 [4.05]	32.25 [9.25]	42.50 [17.45]	**0.006**	**< 0.001**	**0.001**
	2–6 mm annulus	25.90 [3.90]	29.80 [6.38]	35.85 [11.30]	**0.006**	**< 0.001**	**0.002**
	6–10 mm annulus	31.20 [14.45]	44.50 [20.03]	44.30 [24.55]	0.066	**0.008**	0.419
	10–12 mm annulus	43.40 [13.10]	42.75 [19.23]	43.60 [28.20]	0.850	0.743	0.572
	Total diameter	31.40 [6.85]	34.80 [11.38]	43.55 [15.13]	0.05	**< 0.001**	**0.020**
Central layer	0–2 mm annulus	17.60 [1.95]	20.05 [3.38]	21.40 [6.13]	**0.007**	**< 0.001**	**0.033**
	2–6 mm annulus	16.50 [2.25]	17.55 [4.38]	19.55 [4.25]	**0.016**	**< 0.001**	0.123
	6–10 mm annulus	21.50 [12.30]	29.70 [14.15]	28.80 [17.90]	0.088	0.065	0.974
	10–12 mm annulus	29.10 [10.95]	29.70 [10.15]	28.20 [15.35]	0.924	0.707	0.958
	Total diameter	19.50 [6.15]	24.15 [8.25]	25.50 [9.05]	0.061	**0.011**	0.562
Posterior 60 µm	0–2 mm annulus	13.70 [4.50]	16.70 [10.13]	16.55 [8.63]	0.059	**0.006**	0.433
	2–6 mm annulus	14.50 [3.55]	14.40 [9.20]	15.55 [7.68]	0.398	0.089	0.509
	6–10 mm annulus	21.10 [8.20]	21.40 [10.55]	21.05 [11.63]	0.826	0.873	0.948
	10–12 mm annulus	29.20 [9.40]	25.45 [8.18]	24.50 [11.10]	0.210	0.07	0.707
	Total diameter	18.60 [5.50]	18.60 [9.00]	19.05 [8.70]	0.851	0.757	0.769

*P* values in bold indicate statistical significance

*GSU* = gray scale units

Descriptive statistics are presented as median [interquartile range]

### Corneal backscatter in relation to visual acuity

**Fig. 3:**
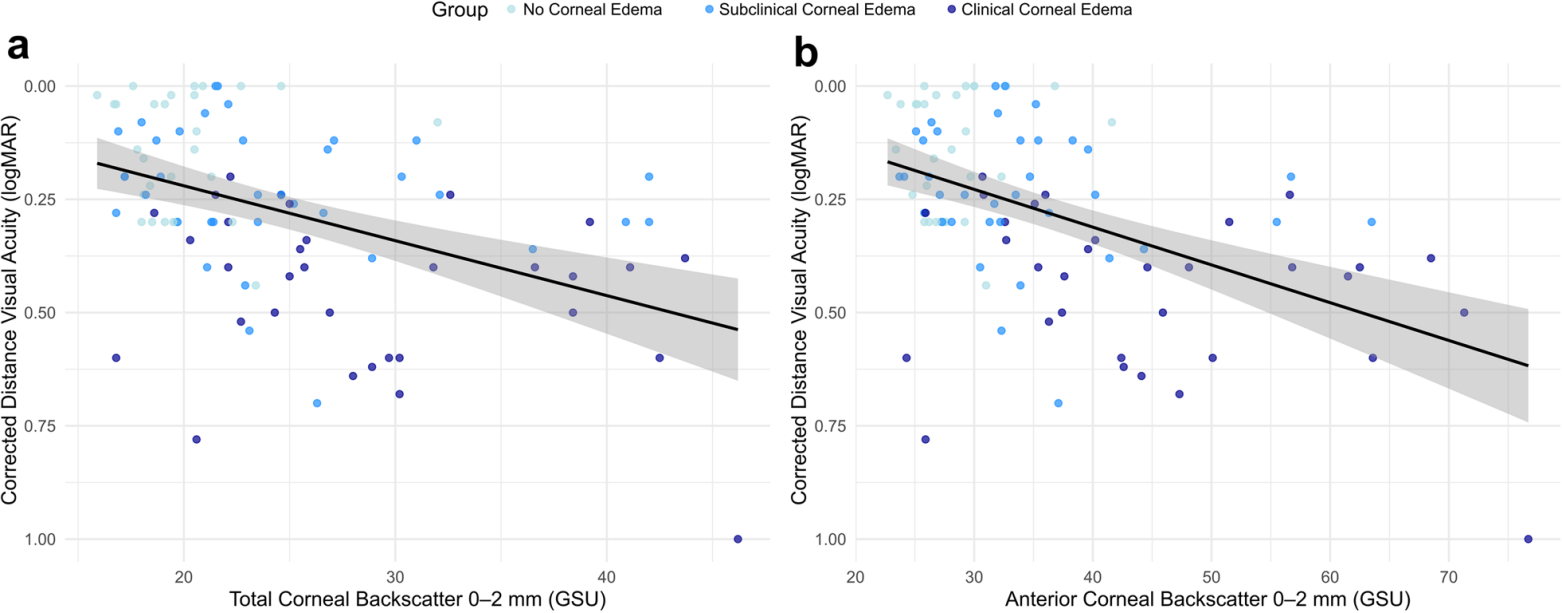
Relationship of total (**a**, β = 0.013, 95% CI: 0.007–0.018; *P* < 0.001, marginal R^2^ = 0.208, Cohen’s f^2^ = 0.26) and anterior corneal backscatter (**b**, β = 0.009, 95% CI: 0.006–0.012; *P* < 0.001, Marginal R^2^ = 0.260, Cohen’s f^2^ = 0.35) in the central 2-mm corneal zone to corrected distance visual acuity. GSU, gray scale units; logMAR, logarithm of the minimum angle of resolution

In addition, we analyzed how corneal backscatter may be a surrogate parameter for visual acuity and found that CDVA significantly correlated with the central corneal backscatter parameters (*P* < 0.05). The total corneal backscatter of the central 2-mm corneal zone showed a statistically significant but moderate correlation to CDVA (β = 0.013, 95% CI: 0.007–0.018; *P* < 0.001, Marginal R^2^ = 0.208, Cohen’s f^2^ = 0.26). However, the corneal backscatter of the anterior 120 µm thick layer in the same zone showed a higher correlation (β = 0.009, 95% CI: 0.006–0.012; *P* < 0.001, Marginal R^2^ = 0.260, Cohen’s f^2^ = 0.35, Fig. 3). Corneal backscatter values showed a higher correlation with CDVA than conventional objective FECD parameters for corneal edema such as TCT (β = 0.001, 95% CI < 0.001–0.002; *P* = 0.004, Marginal R^2^ = 0.096, Cohen’s f^2^ = 0.11), CCT (β = 0.002, 95% CI < 0.001–0.002; *P* < 0.001, Marginal R^2^ = 0.198, Cohen’s f^2^ = 0.25) or corneal back surface asphericity (β = 0.30, 95% CI: 0.135–0.465; *P* < 0.001, Marginal R^2^ = 0.10, Cohen’s f^2^ = 0.11). Only the flat keratometry of the corneal back surface showed a higher correlation with CDVA than corneal backscatter (β = 0.20, 95% CI < 0.13–0.26; *P* < 0.001, Marginal R^2^ = 0.304, Cohen’s f^2^ = 0.44).

### Diagnostic accuracy analysis

To support the diagnosis of subclinical tomographic corneal edema, a diagnostic sensitivity analysis was performed for all corneal backscatter parameters as well as known corneal parameters for FECD assessment such as corneal asphericity (Q value), flat keratometry of the back surface, and CCT.

**Fig. 4:**
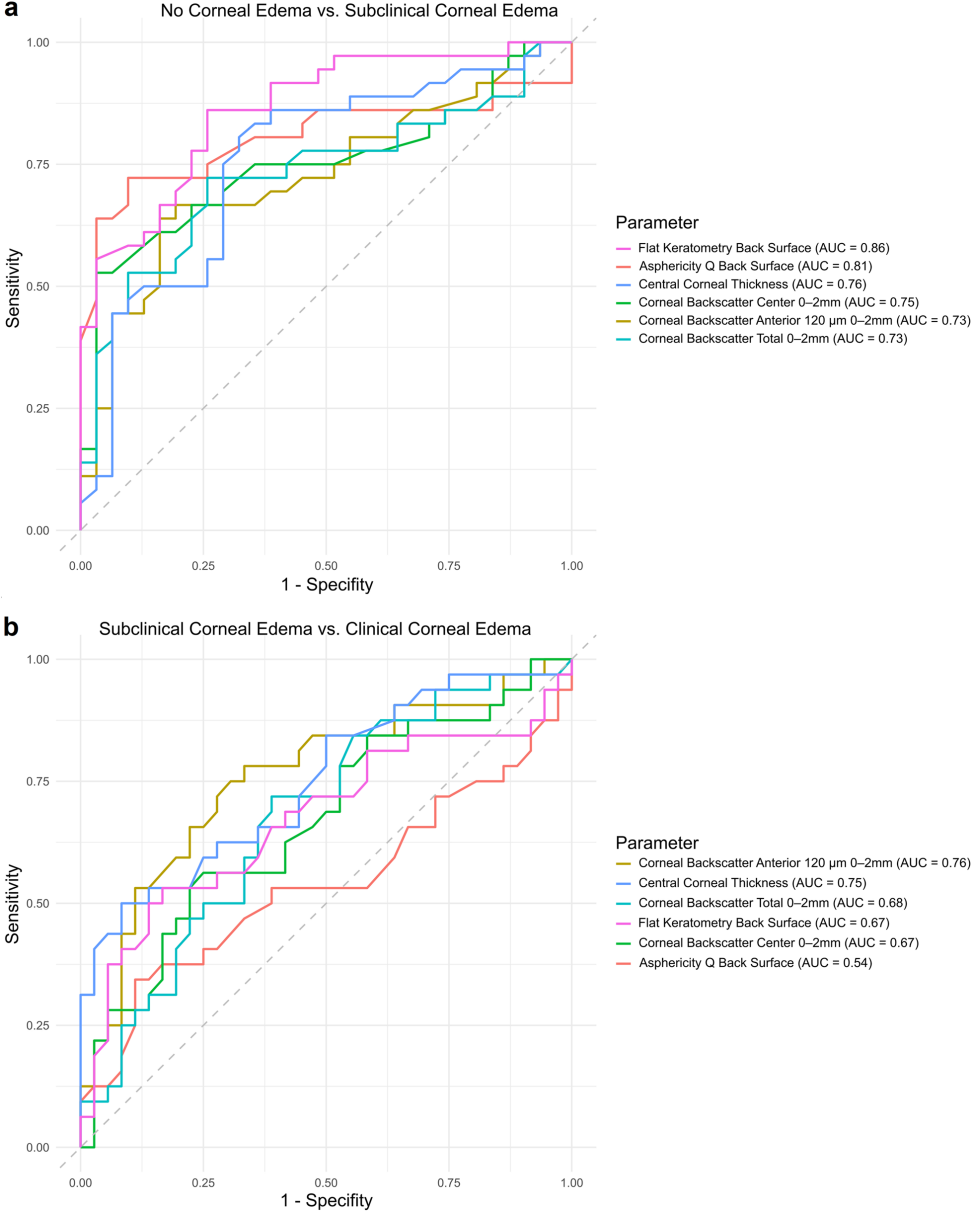
Area under the curve (AUC) of tomographical parameters distinguishing between Fuchs endothelial corneal dystrophy eyes with subclinical corneal edema and without corneal edema (**a**) as well as clinical corneal edema (**b**)

To distinguish between the group without corneal edema and the group with subclinical edema, the parameters with the highest area under the curve (AUC) were the flat keratometry of the corneal back surface (AUC = 0.86, threshold = 6.77 mm, sensitivity = 86.1%, specificity = 74.2%), the asphericity of the corneal back surface (AUC = 0.81, threshold =  − 0.50, sensitivity = 72.2%, specificity = 90.3%), the CCT (AUC = 0.76, threshold = 571.5 µm, sensitivity = 80.6%, specificity = 67.7%), as well as the central corneal backscatter (AUC = 0.75, threshold = 19.8 GSU, sensitivity = 52.8%, specificity = 96.8%), the anterior corneal backscatter (AUC = 0.73, threshold = 31.2 GSU, sensitivity = 63.9%, specificity = 83.9%), and the total corneal backscatter (AUC = 0.73, threshold = 21.0 GSU, sensitivity = 72.2%, specificity = 74.2%, see Fig. 4a) in the central 2-mm zone, respectively. The corneal backscatter of the posterior 60 µm in the central 2-mm zone had a low diagnostic accuracy (AUC = 0.66, threshold = 17.6 GSU, sensitivity = 47.2%, specificity = 90.3%).

To distinguish between the groups with subclinical and clinical corneal edema, the diagnostic potential of the previously mentioned parameters changed with the anterior corneal backscatter performing the best (AUC = 0.76, threshold = 34.9 GSU, sensitivity = 78.1%, specificity = 66.7%), followed by the CCT (AUC = 0.75, threshold = 644.5 µm, sensitivity = 50.0%, specificity = 91.7%) and the total corneal backscatter in the central 2-mm zone (AUC = 0.68, threshold = 23.9 GSU, sensitivity = 71.9%, specificity = 61.1%, see Fig. 4b). The posterior corneal backscatter in the central 2-mm zone could distinguish between both groups even less than the comparison of no edema and subclinical edema (AUC = 0.55, threshold = 14.5 GSU, sensitivity = 81.3%, specificity = 44.4%).

### Multivariate analysis predicting edema status

The explorative multivariate analysis with stepwise backward selection resulted in the exclusion of the central and posterior densitometry of the central 2-mm zone, flat keratometry and asphericity of the corneal back surface, and TCT due to insignificant (*P* > 0.05) contribution to the prediction model. The final model included the anterior densitometry of the central 2-mm zone (Standardized estimate: 0.201, *P* < 0.01) and the CCT (Standardized estimate: 0.611, *P* < 0.001) as significant predictors for edema severity in FECD. The prediction accuracy for edema severity was 54.5% (Table 3).

**Table 3:** Confusion matrix of the generalized estimating equation predicting edema severity

Observed\Predicted	No corneal edema	Subclinical corneal edema	Clinical corneal edema
No corneal edema	8	23	0
Subclinical corneal edema	2	32	2
Clinical corneal edema	1	17	14

## Discussion

In this study, corneal backscatter (measured by Scheimpflug densitometry) significantly increased with edema severity in eyes with FECD. In particular, the anterior 120 µm layer in the central 2-mm zone showed significant correlation with CDVA and was included in the final multivariate analysis model to predict edema group in addition to the CCT. Moreover, corneal backscatter may be helpful as an additional quantitative and objective parameter in addition to the flat keratometry and asphericity of the corneal back surface to differentiate edema severity stages.

The corneal backscatter in FECD patients may increase due to the formation of corneal haze in more advanced stages of the disease [21]. As we have shown in a previous study, patients who underwent DMEK in the clinical edema stage of FECD had worse visual outcomes than those who underwent DMEK in the subclinical edema stage [13]. Additionally, another study found that corneal backscatter may still be elevated after DMEK when operated in the subclinical edema stage compared to healthy controls [22]. The hypothesis for this circumstance may be the formation of subclinical corneal fibrosis due to longer persisting corneal edema, as shown by in-vivo confocal microscopy in advanced bullous keratopathy [23]. This subclinical corneal fibrosis may be measured as corneal backscatter [24], and thus provides insight into the stage of FECD. This hypothesis is supported by the present study showing a positive trend of corneal backscatter and edema severity. Another hypothesis is that increased corneal backscatter reflects corneal edema. In a study by Wacker et al. corneal edema was induced in FECD patients which increased corneal pachymetry as well as corneal backscatter leading to a reduced CDVA [25]. In contrast, some studies have demonstrated that backscatter values remain persistently increased even after successful endothelial keratoplasty, despite complete resolution of corneal edema, when compared with healthy controls [13, 26, 27]. This suggests a more permanent process involved with the development of corneal fibrosis. Future research may identify the exact causes for increased corneal backscatter with progressing FECD stage.

Several studies have shown that corneal backscatter is useful as a surrogate parameter for visual impairment in FECD patients, with higher values indicating worse CDVA [15, 16, 28, 29]. In a cohort of patients with modified Krachmer stages ranging from 1 to 5, Maeno and colleagues showed that CDVA, contrast sensitivity, and disease severity correlate significantly with the central corneal backscatter in all layers [14]. Although our study did not assess contrast sensitivity, we included a larger cohort of patients with both subclinical and clinical edema (modified Krachmer Grade 6), focusing on a more contemporary FECD classification. Moreover, our study shows that central corneal backscatter values correlate better with CDVA than conventional tomographic parameters such as TCT, CCT or corneal asphericity of the back surface. Surrogate parameters for CDVA are particularly important in patients with ocular comorbidities where the reason for visual impairment is difficult to assess. Higher corneal backscatter values may help the decision-making of corneal specialists in regard to endothelial keratoplasty.

A recent study by Poh et al. showed that corneal backscatter values are elevated in FECD eyes with subclinical corneal edema [28]. They found that the corneal backscatter of the anterior 120 µm in the central 2-mm corneal zone yielded the highest accuracy in detecting subclinical corneal edema followed by the central and posterior backscatter values. Another study by Patel et al. found that central anterior and stromal corneal backscatter may help diagnose subclinical edema and predict the improvement in CCT after DMEK [30]. This is in accordance with our study, where more central corneal parameters showed more striking differences between edema groups. The widely established centrifugal disease pattern of FECD, with predominant involvement of the corneal center, aligns well with this observation. However, particularly the anterior densitometry in the central 2-mm zone showed statistically significant predictive value in the multivariate analysis in addition to the CCT, whereas the central and posterior densitometry as well as the flat keratometry and asphericity of the corneal back surface did not contribute significantly to the model and were therefore excluded. Moreover, we found that the posterior backscatter values did not differ as much as the other layers between all three edema severity grades. This may be explained by the predominant edema formation in the anterior layers due to endothelial failure: the remaining functional endothelium may dehydrate the posterior layer while the functional capacity is no longer sufficient for more anterior corneal layers, causing edema and higher backscatter in the anterior layers. On the other hand, Hribek et al. first described a collagen-rich subendothelial fibrillar layer in advanced FECD using elevated values in posterior corneal densitometry confirmed by immunofluorescence staining [31, 32]. These findings contradict the correlations found in the present and previous studies warranting further research with an additional assessment of fibrillar layer presence.

The next generation of corneal imaging incorporating optical coherence tomography may improve diagnostic accuracy due to a higher resolution even in deeper corneal layers [33]. Nonetheless, the study by Poh et al. underlines the trend towards a more objective method to assess FECD staging [28]. Our study corroborates that corneal backscatter may also be used to assess edema severity in FECD patients using the provided thresholds as supportive data in addition to the previously published criteria for diagnosing tomographic corneal edema [11, 34]. The inclusion of objective tomographic parameters such as the anterior corneal backscatter of the corneal center may increase the intra- and inter-observer repeatability for subclinical edema detection [12]. Furthermore, our study found that flat keratometry and asphericity of the corneal back surface showed comparable diagnostic accuracy to central corneal backscatter. The relationships of posterior surface parameters to FECD stage were extensively studied previously with a flatter posterior keratometry correlating to more advanced stages [35, 36]. Yet, these parameters were excluded in a multivariate analysis to predict edema severity. In future, large multifactorial analyses with progression data as well as surgical outcome data are necessary to create a more objective prognosis-relevant staging tool consisting of tomographic biomarkers.

This study is not without limitations. Due to the retrospective study design and the exclusion of patients with ocular comorbidities or insufficient scan quality, selection bias could influence the study results and should be interpreted with caution. Despite the retrospective nature of this study, the secondary exploratory analysis identified the corneal backscatter in the anterior 120 µm layer in the central 2-mm zone to be the most promising biomarker for CDVA impairment and FECD stage. However, larger prospective studies with a primary focus on this parameter are necessary to assess the diagnostic potential for progression prediction and indication for endothelial keratoplasty. Furthermore, biomarkers for FECD should be further analyzed in regard to their correlation to other parameters that reflect visual function, such as contrast sensitivity, straylight, and subjective questionnaires, and in comparison, to an age-matched control group. Age could be a significant confounder as older age may be associated with higher densitometry values [27], but also a higher FECD severity. However, we found no significant correlation between age and corneal backscatter, which is consistent with the findings of Ní Dhubhghaill et al. in the central 6-mm corneal zone.

This study included phakic and pseudophakic patients to reflect the patient distribution in our clinic. As the proportion of pseudophakic patients tended to increase with increasing edema severity, this may confound the exploratory statistical analyses on CDVA correlation. In addition, cataract surgery itself may influence corneal densitometry values, especially in the early postoperative period due to transient corneal edema. However, the literature on this topic is sparse. As our study included only pseudophakic patients who had not undergone cataract surgery within the preceding 3 months, the impact of recent cataract surgery on densitometry is regarded as low; however, it presents a limitation that warrants further investigation. Finally, we included both eyes of some patients in this study, which could confound the statistical analysis due to the possible interdependence of fellow eyes. Yet, we used clustered Wilcoxon rank-sum tests and mixed effects models with patient clustering for the statistical analysis to sufficiently account for the clustered nature of the data [20].

## Conclusion

Corneal backscatter in the central 2-mm zone was significantly elevated with increasing edema severity confirming the diagnostic potential of corneal backscatter in FECD severity grading. Particularly, the anterior corneal layer could be used to differentiate between edema stages in addition to established parameters (e.g., flat keratometry of the posterior corneal surface), which may help corneal specialists in classifying borderline cases. Future studies should evaluate the creation of an objective, progression-relevant scoring system incorporating corneal backscatter as well as other tomographic parameters to classify the FECD stage in more detail.

## Data Availability

The datasets generated and/or analyzed during the current study are not publicly available but are available from the corresponding author on reasonable request.

## Abbreviations

CDVA: Corrected distance visual acuity
CCT: Central corneal thickness
FECD: Fuchs endothelial corneal dystrophy
GSU: Gray scale units
LogMAR: Logarithm of the minimum angle of resolution
TCT: Thinnest corneal thickness
